# Nuclear p16INK4a expression predicts enhanced radiation response in head and neck cancers

**DOI:** 10.18632/oncotarget.9609

**Published:** 2016-05-26

**Authors:** Rüveyda Dok, Layka Abbasi Asbagh, Evert Jan Van Limbergen, Anna Sablina, Sandra Nuyts

**Affiliations:** ^1^ Laboratory of Experimental Radiotherapy, Department of Oncology, KU Leuven, University of Leuven, Leuven, Belgium; ^2^ VIB Center for the Biology of Disease, Leuven, Belgium; ^3^ Department of Human Genetics, KU Leuven, University of Leuven, Leuven, Belgium; ^4^ Department of Radiation Oncology, Leuven Cancer Institute, UZ Leuven, Leuven, Belgium; ^5^ Current address: Maastro Clinic, Maastricht, The Netherlands

**Keywords:** nuclear p16INK4a expression, head and neck cancers, radiotherapy, DNA repair, HPV

## Abstract

Immunohistochemistry analysis of p16INK4a in head and neck squamous cell carcinomas (HNSCC) tumor samples revealed that 28% of tumors showed nuclear/cytoplasmic p16INK4a localization, while 37% of tumors had cytoplasmic p16INK4a. Our previous study showed that p16INK4a inhibits the DNA repair response independently of its function in the cell cycle, suggesting that p16INK4a subcellular localization should be considered during stratification of HNSCC patients.

Using p16INK4a mutants with different localization signals, we found that expression of nuclear p16INK4a, but not cytoplasmic p16INK4a impaired RAD51 foci formation, indicating that nuclear localization of p16INK4a is crucial for its function in DNA repair. We next investigated the role of p16INK4a subcellular localization in radiation response in a retrospective cohort of 261 HNSCC patients treated with chemoradiation. We found that only HNSCC patients expressing nuclear p16INK4a expression showed better outcome, locoregional control and disease free survival, after chemoradiation. In concordance with the patient data, only expression of nuclear p16INK4a increased radiosensitivity of HNSCC cells. These results implicate nuclear p16INK4a expression as a potent marker to predict radiation response of HNSCC patients and should be taken into account in intensification or de-escalation studies.

## INTRODUCTION

Head and neck squamous cell carcinoma (HNSCC) is a heterogeneous disease occurring in different anatomical regions, including the oral cavity, oropharynx, hypopharynx, and larynx [1]. Human papillomavirus (HPV) related head and neck tumors arise primarily in the oropharynx and have a favorable prognosis independently of the treatment modality [1–3]. This indicates that detection of HPV in HNSCC samples can have therapeutic implications, and the most recent American Joint Committee on Cancer (AJCC) staging criteria recommends reporting of HPV status of HNSCC tumors [4, 5].

HPV status in tumors can be assessed by detecting HPV DNA using *in situ* hybridization or polymerase chain reaction (PCR), or by analyzing HPV E6/E7 RNA expression using quantitative reverse transcriptase–PCR (qRT-PCR) [5–8]. However, the detection rates vary across studies partly due to the absence of a consensus on the diagnostic evaluation of HPV in HNSCC [6–8].

HPV status can be also determined indirectly by immunohistochemistry (IHC) analysis of p16INK4a protein expression. p16INK4a is highly expressed in HPV related HNSCC as a consequence of RB inactivation by HPV E7 oncogene [9–13]. In fact, IHC analysis of p16INK4a expression is the most widely used approach to determine HPV status, as the concordance rate in oropharyngeal cancers between HPV direct detection methods and p16INK4a IHC is approximately 90% [6, 8, 14]. However, increased p16INK4a protein expression could be triggered not only by HPV infection, but also by functional loss of RB due to inactivating mutations or chromosomal deletions. The low specificity is referred as one of the major weaknesses of p16INK4a IHC as a surrogate marker for HPV infections [6, 8]. About 10% of the p16INK4a positive tumors show HPV negativity and this is frequently attributed to failing of HPV testing [9, 10, 13].

Multiple recent studies reported that p16INK4a status has a stronger prognostic value compared to HPV in HNSCC [8–10, 13, 15]. Moreover, our recent study revealed that p16INK4a is directly involved in radiation therapy (RT) response by impairing DNA damage response, independently from its role in cell cycle regulation [16]. Therefore, we hypothesized that nuclear p16INK4a localization is crucial for its role in DNA damage response and might be a potent predictor of outcome of HNSCC patients treated with RT. In this study, we evaluated the potential use of nuclear p16INK4a protein expression as a marker for chemoradiation therapy (cRT) response in a retrospectively collected HNSCC patient cohort.

## RESULTS

### Association between p16INK4a localization and patient characteristics

**Figure 1:**
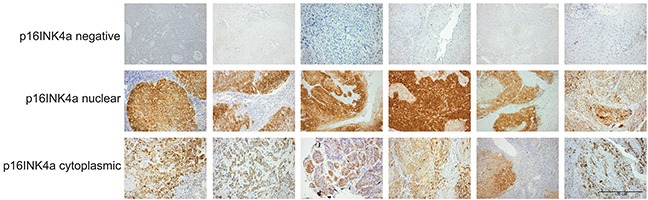
Stratification of oropharyngeal cancer patients according to p16INK4a expression and subcellular localization Examples of pre-treatment biopsies classified as p16INK4a negative (upper panel), nuclear p16INK4a expression (middle panel) and cytoplasmic p16INK4a expression (lower panel). Scale bar, 100μM.

We performed immunochemical analysis of p16INK4a protein expression in pre-treated tumor tissues of 241 patients with oropharyngeal squamous cell carcinoma (OPC) (Figure 1). The baseline patient and tumor characteristics according to p16INK4a expression are summarized in Table 1. Overall, 28% (68 out of 241) of OPC patients showed nuclear p16INK4a expression, while 37% (88 out of 241) showed only cytoplasmic p16INK4a expression, and 35% (85 out of 241) were p16INK4a negative (Table 1). All samples with nuclear p16INK4a staining showed also high cytoplasmic immunostaining (Figure 1). Median age and gender did not differ significantly between these three groups of patients and the vast majority of patients in all groups were diagnosed in disease stage III and IV. No statistically significant difference in the nodal stage, T-stage, tumor localization, treatment modality and radiation dose was noted between different groups. Although the majority of patients had a smoking history, smoking was significantly associated with p16INK4a localization with 54% (13 out of 24) of never smokers showing a nuclear p16INK4a expression.

**Table 1:** Association between patient and tumor characteristics and p16INK4a localization and expression in OPC patients

Patient/Tumor Data	p16		p16		p16				*P*
	Negative		Nuclear		Cytoplasmic		All patients		
	No.	(%)	No.	(%)	No.	(%)	No.	(%)	
**No. of patients**									
	85		68		88		241		
**Gender**									NS^b^
Male	66	78	54	79	73	83	193	80	
Female	19	22	14	21	15	17	48	20	
**Age, years**									NS a
Median (Range)	59	(54-65)	60	(53-66)	56	(50-66)	59	(52-66)	
Nodal stage*									NS^b^
N0/N1	39	46	25	37	39	44	103	43	
N2/N3	46	54	43	63	49	56	138	57	
T stage*									NS^b^
T1/2	27	32	22	33	33	38	82	34	
T3/4	58	68	45	67	54	62	157	66	
Disease stage*									NS^b^
I-II	12	14	3	4	11	12	26	11	
III-IV	73	86	65	96	77	88	215	89	
**Tumor site**									NS^b^
Soft palate	4	5	2	3	1	1	7	3	
Tonsil	27	32	34	50	36	41	97	40	
BOT/vallecula	25	29	25	37	29	33	79	33	
Pharyngeal wall	12	14	5	7	13	15	30	12	
Unknown	17	20	2	3	9	10	28	12	
**HPV**									**<0.0001**^b^
HPV negative	82	97	16	23	67	76	165	69	
HPV positive	1	1	44	65	9	10	54	22	
Unknown	2	2	8	12	12	14	22	9	
**Treatment**									NS^b^
RT	34	40	29	43	34	39	97	40	
RT+CT	49	58	33	49	51	58	133	55	
RT+EGFR inhibitor	2	2	4	6	1	1	7	3	
Unknown	0	0	2	3	2	2	4	2	
**Smoking history**									**0.03**^b^
Never	6	7	13	19	5	6	24	10	
Former	12	14	11	16	10	11	33	14	
Current	62	73	35	51	58	66	155	64	
Unknown	5	6	9	13	15	17	29	12	
**Radiation dose (Gy)**									NS^a^
Median (Range)	68	(66-72)		68	(66-72)	67	(66-72)	67	(67-72)

Abbreviations: NS, not significant; BOT, base of tongue; RT, radiotherapy; CT, chemotherapy; EGFR, epidermal growth factor

* International Union of Cancer Research 1982 classification; *P* was determined by

a ANOVA;

b chi square test.

To assess the correlation between p16INK4a localization and HPV, we determined HPV status in 219 patients out of 241 patients, as 22 patients had insufficient tumor material for HPV testing. Of these, 54 (25%) were HPV positive and 165 (75%) were HPV negative. Strikingly, we observed a strong correlation (phi coefficient: 0.70) between HPV and p16INK4a nuclear localization as 81% (44 out of 54) of HPV positive patients also had nuclear p16INK4a expression and only 17% (9 out of 54) of HPV positive patients had cytoplasmic localization of p16INK4a.

### Survival outcomes based on p16INK4a expression and localization

**Figure 2:**
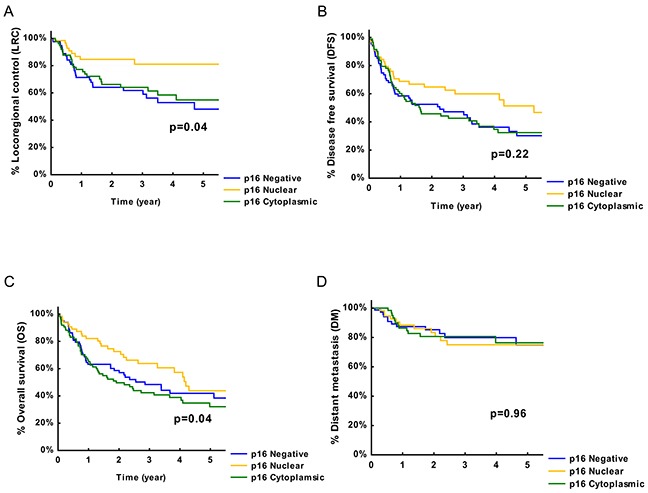
Association between p16INK4a and survival outcome **A.** Survival of HNSCC patients with different status of p16INK4a expression presented by Kaplan-Meier curves with locoregional tumor control (LRC) as end-point. **B.** Survival of HNSCC patients with different status of p16INK4a expression presented by Kaplan-Meier curves with disease free survival (DFS) as end-point. **C.** Survival of HNSCC patients with different status of p16INK4a expression presented by Kaplan-Meier curves with overall survival (OS) as end-point. **D.** Survival of HNSCC patients with different status of p16INK4a expression presented by Kaplan-Meier curves with distant metastasis (DM) control as end-point. P values are determined by log-rank tests.

We next examined the association between p16INK4a subcellular localization and outcome parameters such as locoregional tumor control (LRC), disease free survival (DFS) and overall survival (OS). Median follow-up was 4.24 years (lower quartile: 2.34 year; upper quartile: 6.45 year). We found significantly better LRC rates in nuclear p16INK4a expressing patients (5-year LRC rates: 80%; P=0.04) compared to p16INK4a negative (5-year LRC rates: 50%) or cytoplasmic p16INK4a expressing (5-year LRC rates: 58%) patients (Figure 2A).

Moreover, univariable analysis revealed that nuclear p16INK4a expression (HR: 0.35; 95% CI: 0.17-0.76; P=0.007), but not cytoplasmic p16INK4a expression (HR: 0.85; 95%CI: 0.65-1.46 P=0.23) resulted in a lower risk for locoregional failure (Table 2). In addition to p16INK4a expression pattern, T-stage and HPV status were significantly associated with better LRC (Table 2).

**Table 2:** Effect of patient and tumor characteristics on LRC, DFS and OS

	LRC			DFS			OS		
	HR	95% CI	*P*	HR	95% CI	*P*	HR	95% CI	*P*
**p16INK4a**									
Cytoplasmic	0.85	0.51-1.47	NS	0.98	0.65-1.46	NS	1.21	0.81-1.83	NS
Nuclear	0.35	0.17-0.76	**0.007**	0.63	0.40-1.02	NS	0.73	0.45-1.18	NS
Negative	1 (ref)			1 (ref)			1 (ref)		
**Gender**									
Male	0.99	0.50-1.86	NS	0.99	0.63-1.54	NS	1.76	1.04-2.97	**0.03**
Female	1 (ref)			1 (ref)			1 (ref)		
Nodal stage*									
N0-N1	1.20	0.74-1.95	NS	1.23	0.87-1.74	NS	1.04	0.74-1.48	NS
N2-N3	1 (ref)			1 (ref)			1 (ref)		
T-stage*									
T1-2	0.54	0.31-0.93	**0.025**	0.48	0.32-0.71	**<0.0001**	0.42	0.28-0.63	**<0.0001**
T3-4	1 (ref)			1 (ref)			1 (ref)		
Disease stage*									
I-II	1.54	0.76-3.1	NS	1.46	0.86-2.47	NS	0.96	0.52-1.78	NS
III-IV	1 (ref)			1 (ref)			1 (ref)		
**Tumor site**									
Soft palate	0.30	0.03-2.39	NS	0.34	0.08-1.46	NS	0.36	0.08-1.56	NS
Tonsil	0.74	0.36-1.51	NS	0.64	0.38-1.10	NS	0.77	0.45-1.32	NS
BOT/vallecula	0.62	0.28-1.33	NS	0.89	0.52-1.52	NS	0.92	0.53-1.60	NS
Pharyngeal wall	1 (ref)			1 (ref)			1 (ref)		
**HPV**									
Positive	0.35	0.16-0.77	**0.009**	0.52	0.32-0.86	**0.009**	0.56	0.34-0.90	**0.02**
Negative	1 (ref)			1 (ref)			1 (ref)		
**Smoking history**									
Former	0.75	0.19-2.99	NS	1.24	0.50-3.03	NS	0.98	0.41-2.38	NS
Current	2.40	0.86-6.67	NS	2.37	1.15-4.89	**0.02**	2.05	1.03-4.07	**0.04**
Never	1 (ref)			1 (ref)			1 (ref)		

Abbreviations: NS, not significant; BOT, base of tongue; HR, hazard ratio; CI, confidence interval; ref, reference; LRC, locoregional control; DFS, disease free survival; OS, Overall survival;

* International Union of Cancer Research 1982 classification;

*P* was determined by cox-regression analysis

Although, the different p16INK4a expressing groups did not show significant differences in DFS rates, nuclear p16INK4a expressing patients showed a 5 year DFS rates of 52% compared to p16INK4a negative and cytoplasmic p16INK4a positive patients with 5 year DFS rates of 32% and 30%, respectively (Figure 2B). Furthermore, nuclear p16INK4a positive patients showed 5-year OS rates of 44% (P=0.04) compared to cytoplasmic p16INK4a positive or p16INK4a negative HNSCC patients with respective 5-year OS rates of 35% and 42% (Figure 2C). However, no significant difference in distant metastasis (DM) control rates was observed between the groups (Figure 2D). Comparable with the survival curves univariable analysis showed no significant association between p16INK4a expression and OS (HR: 0.73; 95% CI: 0.45-1.18; P=0.19) and a trend to significance with DFS (HR: 0.63; 95% CI: 0.40-1.02; P=0.06).

The effect of p16INK4a localization on clinical outcome was further studied after adjustment for known prognostic factors [2, 9], including gender, age, nodal and T-stage, smoking history and tumor localization (Table 3). The adjusted HR ratios for nuclear p16INK4a expression were 0.25 (95% CI: 0.09-0.66; P=0.005) and 0.50 (95% CI: 0.29-0.98 P=0.01) for LRC and DFS, respectively. However, multivariate analysis did not show a significant effect of nuclear p16INK4a expression on OS (HR: 0.62 95% CI: 0.35-1.12; P=0.12). Taken together, these results indicate that nuclear p16INK4a expression is a strong predictive factor for LRC after adjustment for other known clinical parameters, suggesting a crucial role for nuclear p16INK4a expression in radiation treatment response and local control of HNSCC patients.

**Table 3:** Multivariate analysis of LRC, DFS and OS

	LRC			DFS			OS		
	HR	95% CI	*P*	HR	95% CI	*P*	HR	95% CI	*P*
**p16INK4a**									
Cytoplasmic	0.68	0.35-1.28	NS	0.75	0.47-1.21	NS	1.00	0.61-1.65	NS
Nuclear	0.25	0.09-0.66	**0.005**	0.50	0.29-0.89	**0.01**	0.62	0.35-1.12	NS
Negative	1 (ref)			1 (ref)			1 (ref)		
**Gender**									
Male	0.58	0.28-1.19	NS	0.85	0.48-1.51	NS	1.66	0.85-3.22	NS
Female	1 (ref)			1 (ref)			1 (ref)		
**Age**									
continuous	1.02	0.98-1.05	NS	1.03	1.01-1.06	**0.01**	1.02	0.99-1.04	NS
Nodal stage*									
N0-N1	0.70	0.34-1.45	NS	0.99	0.60-1.62	NS	0.77	0.46-1.27	NS
N2-N3	1 (ref)			1 (ref)			1 (ref)		
T stage*									
T1-2	0.52	0.25-1.05	NS	0.47	0.28-0.80	**0.004**	0.35	0.20-0.61	**0.0001**
T3-4	1 (ref)			1 (ref)			1 (ref)		
**Tumor site**									
Soft palate	0.19	0.02-1.89	NS	0.33	0.06-1.62	NS	0.75	0.15-3.79	NS
Tonsil	0.69	0.30-1.60	NS	0.80	0.43-1.50	NS	1.12	0.60-2.10	NS
BOT/vallecula	0.59	0.24-1.46	NS	0.98	0.52-1.86	NS	1.00	0.52-1.92	NS
Pharyngeal wall	1 (ref)			1 (ref)			1 (ref)		
**Smoking history**									
Former	0.49	0.11-2.17	NS	0.82	0.32-2.15	NS	0.67	0.26-1.75	NS
Current	0.67	0.36-1.28	NS	1.57	0.71-3.47	NS	1.17	0.54-2.56	NS
Never	1 (ref)			1 (ref)			1 (ref)		

Abbreviations: NS, not significant; BOT, base of tongue; HR, hazard ratio; CI, confidence interval; ref, reference; LRC, locoregional control; DFS, disease free survival; OS, Overall survival;

* International Union of Cancer Research 1982 classification.

*P* was determined by cox-regression analysis

In addition, the influence of HPV status on LRC was evaluated in nuclear p16INK4a and cytoplasmic p16INK4a expressing patients. No significant differences were found (cytoplasmic p16INK4a expression HR: 0.43; 95% CI: 0.10-1.85 P=0.26 and nuclear p16INK4a expression HR: 0.50; 95% CI: 0.14-1.90 P=0.31). This result indicates that the high concordance between HPV status and nuclear p16INK4a expression will make it difficult to distinguish between the effects of nuclear p16INK4a expression and HPV status.

### Nuclear localization of p16INK4a is essential for its DNA repair function in HNSCC cells

Our previous study demonstrates that p16INK4a overexpression results in an impaired homologous recombination DNA repair (HRR) response and decreased cell survival after RT independently of its function in cell cycle regulation [16]. Given that our data strongly suggest that nuclear localization of p16INK4a could predict LRC rates, we further elucidated the effect of p16INK4a subcellular localization on RT response in HNSCC cells.

**Figure 3:**
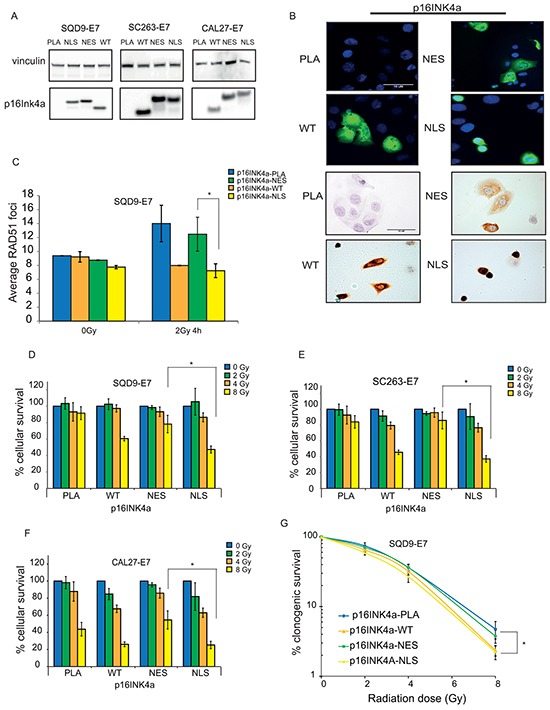
The effect of p16INK4a nuclear localization on radiation response in HNSCC cells **A.** Immunoblot analysis of expression of p16INK4a fused with different localization signals in the indicated cell lines. **B.** Immunofluorescence (upper panel) and immunocytochemistry analysis (lower panel) of p16INK4a expression and localization in SQD9-E7 cells after overexpression of the indicated constructs. Scale bar, 10μM. **C.** RAD51 foci formation SQD9-E7 expressing the indicated constructs 4 hours after treatment with 2Gy ionizing radiation. The result is shown as mean ± SEM of two experiments. **D-F.** Cell survival of HNSCC cells expressing the indicated p16INK4a constructs upon ionizing radiation as detected by sulforhodamine B assay. **G.** Clonogenic survival assay of SQD9-E7 cells expressing WT-p16INK4a, p16INK4a-NLS, p16INK4a-NES, or an empty vector (PLA) treated with the indicated doses of ionizing radiation. (D-G) Cell survival is expressed as ± s.e.m. relative to non-irradiated cells from at least two experiments. PLA: empty vector; WT: wild-type; NES: nuclear export signal; NLS: nuclear localization signal. P-values are calculated by two-sided t-test.

Because p16INK4a does not have a recognizable nuclear localization signal (NLS) or a nuclear export signal (NES), we generated expression constructs encoding p16INK4a fused with either the HIV Rev NES, or Simian Virus 40 T antigen NLS. Immunoblotting analysis confirmed that expression levels of the fused p16INK4a proteins were comparable to expression levels of the wild-type (WT) protein (Figure 3A). Moreover, immunofluorescence and immunocytochemistry analyses showed that WT-p16INK4a was found in both nucleus and cytoplasm. On the other hand, p16INK4a-NLS was observed mostly in the nucleus, whereas p16INK4a-NES was localized in the cytoplasm (Figure 3B).

We next overexpressed the p16INK4a proteins in HPV-negative/p16INK4a-negative HNSCC cells. To exclude any possible effect of p16INK4a on cell cycle, we also inhibited the RB pathway by introducing HPV-E7 oncogene in all our cell lines.

To assess whether subcellular localization of p16INK4a affects HRR response, we examined RAD51 foci formation in SQD9-E7 cells expressing the generated p16INK4a constructs. In line with our previous finding [16], overexpression of WT-p16INK4a led to impaired RAD51 foci formation upon IR, confirming the contribution of p16INK4a to HRR DNA repair mechanism (Figure 3C). Similar to WT-p16INK4a, p16INK4a-NLS overexpressing cells also showed a decreased number of RAD51 foci after IR, whereas p16INK4a-NES overexpression did not affect RAD51 foci formation, indicating that nuclear localization of p16INK4a is necessary for its function in DNA repair (Figure 3C).

We then elucidated the effect of subcellular p16INK4a localization on survival after RT using short-term survival assays. We introduced WT-p16INK4a, p16INK4a-NLS, or p16INK4a-NES into SQD9, CAL27, and SC263 cells overexpressing HPV-E7. In concordance with patient data, we found that nuclear p16INK4a overexpression increased radiation sensitivity, while cytoplasmic p16INK4a did not affect cell survival upon irradiation (Figure 3D–3F). These findings were further confirmed by a clonogenic assay. Only cells expressing either WT-p16INK4a, or p16INK4a-NLS showed higher radiation sensitivity when compared to cells expressing p16INK4a-NES. These results further confirm the importance of p16INK4a nuclear localization in RT response (Figure 3G).

## DISCUSSION

The prognostic value of p16INK4a as a surrogate marker for HPV infections in OPCs is documented in several pivotal studies. However, the prognostic and biological relevance of p16INK4a as a biomarker independent of HPV infection is still highly debated and worth further investigation [6–8, 14]. Our recent study strongly reveals that p16INK4a decreased the DNA repair independently of its functions in cell cycle control. This suggests the importance of subcellular localization of p16INK4a in risk stratification of HNSCC patients [16–18].

Our data clearly demonstrate the importance of patient stratification according to p16INK4a subcellular localization. Patients with nuclear p16INK4a expression showed a significant reduction in risk for locoregional failure and disease specific failure. Importantly, nuclear p16INK4a expression was the only predictive factor for LRC after adjustment for other known clinical parameters and did not reduce the risk for OS, suggesting a predominant role for nuclear p16INK4a expression in radiation treatment response and local control. Previously Zhao et al. also reported that differences in p16INK4a localization could affect survival outcomes in a mixed retrospective analysis of OP and non-OPC tumors. However, no firm conclusions were made in this study due to the small cohort size [19].

Despite our primary aim to assess the correlation between p16INK4a expression and subcellular localization and clinical outcome, the confounding effect of HPV could not be overlooked. As in previous studies [6, 8, 14], the high correlation between HPV positivity and nuclear p16INK4a expression makes it difficult to separate the effects p16INK4a expression and HPV infections.

Nonetheless, we found that nuclear p16INK4a modulates RT response by reducing the HRR activity. In line with our retrospective analysis, nuclear p16INK4a but not cytoplasmic p16INK4a inhibits DNA repair and sensitizes the cells to RT. The absence of a radiation sensitizing effect of cytoplasmic p16INK4a is in agreement with previous reports in other cancer types, where cytoplasmic p16INK4a expression is associated with worse patient survival [19–21].

In conclusion, our study clearly demonstrates that nuclear p16INK4a, but not cytoplasmic, expression results in better outcome of HNSCC patients confirming the importance of nuclear p16INK4a localization in DNA repair and RT response. These results suggest that nuclear p16INK4a expression can be used as a standalone marker for prediction of radiation sensitivity in HNSCC patients.

## MATERIALS AND METHODS

### Study cohort

The study cohort consisted out of 261 patients with oropharyngeal squamous cell carcinoma (OPC), who were diagnosed between 2000 and 2010. For 241 patients paraffin-embedded formalin fixed pre-treatment (FFPE) tumor tissues were available. The human tumor samples were acquired according protocols approved by the Ethical board of the University Hospitals Leuven (Leuven, Belgium). Former smokers are defined as patient who stopped smoking longer than a year ago before the date of diagnosis.

### p16INK4a immunohistochemistry analysis and HPV detection

IHC for p16INK4a (G175-405, BD Pharmingen) expression was performed as previously described [16, 22]. Sections of p16INK4a cervical carcinoma were used as positive controls.

P16INK4a expression was scored according to staining intensity and percentage positive tumor cells. Tumors were classified as nuclear p16INK4a expressing, cytoplasmic p16INK4a expressing, and p16INK4a negative groups. The nuclear p16INK4a expressing group was defined as nuclear p16INK4a expression in >10% of the carcinoma cells. The cytoplasmic p16INK4a expressing group was defined as only cytoplasmic p16INK4a expression in >10% of carcinoma cells. The p16INK4a negative group was defined as <10% p16INK4a nuclear and/or cytoplasmic staining of carcinoma cells [23, 24]. HPV status was determined by HPV based GP5+/6+ PCR as previously described [22].

### Cell lines and reagents

The HPV/p16INK4a negative SQD9, SC263 and CAL27 cell lines, a generous gift of Dr. A. Begg, the Netherlands Cancer Institute (Amsterdam; the Netherlands), were cultured as previously described [16]. The cells were transfected with p16INK4a-nuclear localization signal (NLS: PKKKRKV), p16INK4a-nuclear export signal (NES: LPPLERLTL), WT-p16INK4a plasmid sequences and pBabe HPV-E7 (a generous gift of Dr. K. Munger; Harvard; USA) using lipofectamine 2000 (Life technologies) according to the manufacturers protocol. p16INK4a constructs were generated by PCR amplification (NES: 5′-ATTGTCGACTCACAG GGTCAGTCTCTCCAGAGGAGGCAGATCGGGGATGTCTGA-3′; NLS: 5′-ATTGTCGACTTA AACCTTACGCTTCTTCTTTGGATCGGGGATGTCTGA-3′). DNA damage was induced by ionizing radiation (199 kV, balthograph Baltho).

### Colony formation and cell viability

48 hours after transfection, cells were exposed to increasing dose of ionizing radiation (0-8Gy) and plated into 10 cm dishes. After 2 to 3 weeks cells were fixed with 2.5% glutaraldehyde in PBS and stained with 0.2% crystal violet. The colonies containing 50 cells or more were counted with ColCount colony counter (Oxford Optronix). Survival fractions were corrected for the plating efficiencies. For cell viability assay, transfected cells were seeded with 20% confluence on 96-well plates and after 7 days a short-term survival assay (sulforhodamine B assay) was performed as previously described [16].

### Immunoblotting, immunocytochemistry and immunofluorescence analyses of p16INK4a

For immunoblotting, cells were lysed with RIPA buffer containing protease and phosphatase inhibitors (Roche). Proteins were subjected to SDS-PAGE. Immunoblotting was performed with antibodies against vinculin (clone hVIN-1, Sigma-Aldrich), p16INK4a (clone G175-405, BD Pharmingen).

For immunocytochemistry (ICC) and immunofluorescence (IF) analyses, cells were seeded on coverslips and fixed with ice cold methanol at −20°C for 15 minutes. Cells were then incubated with anti-p16INK4a antibody overnight at 4°C for ICC and 1 hour at room temperature for IF. Secondary antibodies with HRP or FITC conjugated were incubated and cells were analyzed using light microscope (Olympus) or with a bright-field immunofluorescence microscope (Zeiss). RAD51 (clone 14B4, Novus Biologicals) foci formation was stained and analyzed by in cell analyzer (BD Biosciences) as previously described [16, 25].

### Statistical analysis

Differences between p16INK4a negative, p16INK4a cytoplasmic, and p16INK4a nuclear expressing groups were analyzed using the Chi-square test in case of categorical predictors whereas the one-way analysis of variance was used in cases of continuous predictors. The phi coefficient was used to assess the association between p16 localization and HPV status.

Survival rates were estimated by Kaplan-Meier method and compared with a log-rank test. Univariable and multivariate hazard ratios (HR) and confidence intervals (CI) were estimated using Cox proportional hazard models. For *in vitro* cell survival analysis Student t-test was used. All statistical analyses were performed 2 sided and were considered statistically significant for p≤0.05.
